# Response to Cold: A Comparative Transcriptomic Analysis in Eight Cold-Adapted Yeasts

**DOI:** 10.3389/fmicb.2022.828536

**Published:** 2022-02-23

**Authors:** Marcelo Baeza, Sergio Zúñiga, Vicente Peragallo, Fernando Gutierrez, Salvador Barahona, Jennifer Alcaino, Víctor Cifuentes

**Affiliations:** ^1^Departamento de Ciencias Ecológicas, Facultad de Ciencias, Universidad de Chile, Santiago, Chile; ^2^Centro de Biotecnología, Facultad de Ciencias, Universidad de Chile, Santiago, Chile

**Keywords:** cold-adapted yeasts, Antarctic yeasts, transcriptomes, cold adaptation, stress genes, codon bias

## Abstract

Microorganisms have evolved to colonize all biospheres, including extremely cold environments, facing several stressor conditions, mainly low/freezing temperatures. In general, terms, the strategies developed by cold-adapted microorganisms include the synthesis of cryoprotectant and stress-protectant molecules, cold-active proteins, especially enzymes, and membrane fluidity regulation. The strategy could differ among microorganisms and concerns the characteristics of the cold environment of the microorganism, such as seasonal temperature changes. Microorganisms can develop strategies to grow efficiently at low temperatures or tolerate them and grow under favorable conditions. These differences can be found among the same kind of microorganisms and from the same cold habitat. In this work, eight cold-adapted yeasts isolated from King George Island, subAntarctic region, which differ in their growth properties, were studied about their response to low temperatures at the transcriptomic level. Sixteen ORFeomes were assembled and used for gene prediction and functional annotation, determination of gene expression changes, protein flexibilities of translated genes, and codon usage bias. Putative genes related to the response to all main kinds of stress were found. The total number of differentially expressed genes was related to the temperature variation that each yeast faced. The findings from multiple comparative analyses among yeasts based on gene expression changes and protein flexibility by cellular functions and codon usage bias raise significant differences in response to cold among the studied Antarctic yeasts. The way a yeast responds to temperature change appears to be more related to its optimal temperature for growth (OTG) than growth velocity. Yeasts with higher OTG prepare to downregulate their metabolism to enter the dormancy stage. In comparison, yeasts with lower OTG perform minor adjustments to make their metabolism adequate and maintain their growth at lower temperatures.

## Introduction

Microorganisms inhabiting cold environments, which are predominant on our planet and are defined as having constant temperatures below 5°C (Siddiqui et al., 2013; Buzzini and Margesin, 2014; Margesin, 2017), must face several stressor conditions, related mainly to low/freezing temperatures. A general consequence of low temperature is a slow reaction rate; consequently, microorganisms living in cold environments have evolved to function efficiently at low temperatures by molecular and metabolic adaptations. Among the mechanisms described to counteract low temperatures and freezing, there is the synthesis of cryoprotectant molecules, cold-active enzymes, and membrane fluidity regulation (Margesin et al., 2007; Alcaíno et al., 2015; Baeza et al., 2017). In the few sequenced genomes of cold-adapted bacteria, the most studied cold-adapted microorganism (Demain and Adrio, 2008; Margesin and Feller, 2010), the genes involved in protein synthesis have attracted attention. The higher number of rRNA and tRNA genes observed in cold-adapted than mesophilic bacterial genomes was suggested as a compensation for the reduced translation rate at low temperature (Methé et al., 2005; Médigue et al., 2005; Riley et al., 2008), a process crucial for rapid metabolism and response to environmental changes. At the protein level, a reduced proline content has been described in bacterial cold-active proteins and suggested as an adaptation to attenuate the negative effect of proline isomerization on protein folding (Wedemeyer et al., 2002; Feller, 2003, 2010). The active sites of cold-active enzymes have been described as more flexible and voluminous than their mesophilic counterparts, being, in turn, their most heat-labile structural element (Jung et al., 2008; Feller, 2013; Parvizpour et al., 2021; Yusof et al., 2021).

Regarding studies of microbial response to cold stress, most used mesophilic models, such as *Saccharomyces cerevisiae* (Schade et al., 2004), *Escherichia coli* (Mihoub, 2003), and *Bacillus subtilis* (Budde et al., 2006), report mainly the inhibition of protein synthesis, induction of cold-shock proteins, (Jones et al., 1987; Willimsky et al., 1992; Goldstein, 2007), accumulation of compatible solutes, increase of unsaturated fatty acids in cell membranes, and antioxidant response (Borges et al., 2002; Zhang et al., 2003; Chattopadhyay, 2006; Casanueva et al., 2010).

In recent years, studies using microorganisms isolated from cold regions have been performed, obtaining interesting results. In a transcriptomic comparison of the Antarctic yeast *Rhodotorula frigidialcoholis* cultivated at 0°C vs. 23°C, the downregulation of genes involved in the electron transport chain and citrate cycle suggested a switch from respiratory to fermentative metabolism. Furthermore, the increase in diversity and abundance of sRNAs and the Dicer gene was suggested as a novel mechanism of adaptation through the downregulation of translation processes (Touchette et al., 2021). In cold-shock response studies of three species of psychrotrophic lactic acid bacteria, the main and common induced gene encoded cold-shock protein A. Furthermore, the genes encoding some ribosomal proteins, tRNA modification, rRNA modification, ABC and efflux MFS transporter genes were upregulated (Duru et al., 2021). In a comparative study of the Antarctic bacterium *Psychrobacter* sp. PAMC 21119 grown at –5 and 20°C genes such as for translation, ribosomal structure, and biogenesis were upregulated at subzero temperature, and genes related to lipid transport and metabolism were downregulated at the transcriptomic level. Proteins involved in metabolite transport, protein folding, and membrane fluidity were the most prominently upregulated at cold in proteomic analysis. An interesting finding was an isoform exchange of cold-shock proteins that may be an adaptation to catalyze similar reactions at different growth temperatures (Koh et al., 2017).

We isolated and characterized yeasts from soils of several Antarctic regions (Carrasco et al., 2012; Barahona et al., 2016; Troncoso et al., 2017), which displayed significant differences in the optimal temperatures for growth and activity of their secreted hydrolytic enzymes, such as amylases, cellulases, and glucose oxidases (Carrasco et al., 2016, 2017a,b). The draft genomes were determined for eight of these Antarctic yeasts ranging from 10.7 Mb (*Tetracladium* sp.) to 30.7 Mb (*Leucosporidium creatinivorum*), and the GC content ranged from 37% (*Candida sake*) to 60% (*L. creatinivorum*) (Baeza et al., 2021). Most predicted orthologous genes related to stress responses corresponded to oxidative, general, cold, and osmotic stress. In a comparative analysis of the amino acid compositions of their *in silico* translated proteins concerning yeast growth parameters, more similarities were found among yeasts growing slowly or having low optimal temperatures for growth. Instead, an inverse correlation between the content of flexible amino acids and the optimal temperature for growth was found for yeasts growing faster.

This study analyzed the gene expression changes in eight Antarctic yeasts when their incubation temperatures were switched from regular to 4°C. Putative genes were predicted from their ORFeomes and functionally classified; their expression level and codon usage bias were determined and characterized according to the estimated flexibility of encoding proteins. Both up- and downregulated genes were found, including those canonically associated with the response to all main kinds of stress, whose number was directly related to the temperature variation that each yeast faced. Multiple comparative analyses were performed based on gene expression changes, protein flexibility, cellular functions, and codon bias. From the findings obtained, yeasts isolated from the same Antarctic region could be stated to have developed different strategies to respond to cold.

## Materials and Methods

### Yeasts and Strains, Culture Conditions, and Molecular Procedures

The yeasts studied were isolated from soil samples of King George Island (Carrasco et al., 2012) and were routinely grown in yeast medium (YM) supplemented with 1% glucose and 1.5% agar for semisolid media. DNA purification, quantification and characterization, electrophoresis, and spectroscopic techniques were undertaken according to standardized methodologies (Sambrook and Russell, 2001). Procedures with commercial kits were performed according to the manufacturer’s instructions.

### RNA-seq and Basic Bioinformatics Analysis

Yeasts were grown in 200 ml YM broth medium, shaken at 160 rpm at different temperatures: *Mrakia gelida* T29_3-4C (10°C); *Cryptococcus* sp. T15-22C (10°C); *Vishniacozyma victoriae* T18_1-22C (15°C); *Phenoliferia glacialis* T8_3-4C (15°C); *Leucosporidium creatinivorum* T33_1-10C (22°C); *Candida sake* H14_1-4C (22°C); *Tetracladium sp.* T11_2-10C (22°C); *Wickerhamomyces anomalus* H01_1-10C (30°C), henceforth called high temperature (HT). Once a midlog growth phase was reached, half of each culture was transferred to a new sterile flask; one was incubated at the same temperature, and the other was incubated at 4°C for 6 h. The cellular pellets were obtained from each yeast culture by centrifugation at 7,000 *g* at 4°C for 10 min, resuspended in 5 ml of water, centrifuged at 7,000 *g* at 4°C for 10 min, frozen at –80°C, and sent to Omics2view Consulting company (Schauenburgerstrasse, Germany) for RNA purification using TRIzol (Invitrogen), sequencing using the Illumina HiSeq 4000, 2 × 150 bp, and bioinformatics processing as follows. Quality of demultiplexed reads was checked with FastQC v0.11.7 (Andrews, 2018) and a summary QC report created with MultiQC v1.7 (Ewels et al., 2016) (Supplementary Table 1). Read quality trimming was performed with the BBTools package v38.19 (Bushnell, 2019). This comprised the removal of optical duplicates, adapter sequences, low-entropy reads, and trimming of bases with quality scores <20. Reads with invalid or ambiguous bases and reads with a length <127 base pairs (bp) were discarded. Only reads surviving quality trimming as pairs entered downstream analysis. Read quality recalibration and error correction was performed with the BBTools package v38.19 (Bushnell, 2019). Quality-trimmed reads were aligned to a preliminary *de novo* assembly made with Tadpole from a subset of the quality-trimmed reads. Alignment information was used to recalibrate the base quality of all quality-trimmed reads. Sequencing errors were corrected by consecutively applying BBTools programs BBMerge, Clumpify, and Tadpole in error correction mode on the quality-recalibrated reads. Results are referred to as “filtered reads.” A total of 31-bp kmers of filtered reads were normalized with BBNorm to a target kmer depth of 100×, with a minimum kmer depth of 3×. An assembly of these normalized reads was constructed for each individual sample with rnaSPAdes v3.13.0 (Bankevich et al., 2012). Scaffolds produced by rnaSPAdes were merged with Dedupe from the BBTools package v38.19 (Bushnell, 2019) if they exhibited a minimum similarity of 99% and were filtered for a minimum length of 240 bp. The result is referred to as “primary assembly.” Filtered reads were back-mapped onto the primary assembly with BBMap from the BBTools package v38.19 (Bushnell, 2019). For each contig in the primary assembly, the overall coverage was determined from the number of unambiguously mapped reads. Contigs of length ≥240 bp and coverage ≥10× were considered reliable and are referred to as “filtered assembly.” Data were uploaded to the NCBI database Bioprojects: *Cryptococcus* sp., PRJNA761919; *C. sake*, PRJNA761920; *L. creatinivorum*, PRJNA761921; *M. gelida*, PRJNA761923; *P. glacialis*, PRJNA761924; *Tetracladium* sp., PRJNA783217; *V. victoriae*, PRJNA761926; and *W. anomalus*, PRJNA761928.

### Prediction, Functional Annotation, Expression Level Determination, and Comparative Analysis of ORFs

Analysis was performed using Geneious Prime version 2020.1.2 software (Kearse et al., 2012) and the included plugins, such as ORF finder, Blast (ncbi-blast-2.8.1+), and Geneious RNAseq. ORFs with lengths ≥210 nt were predicted, translated, and compared by Blastp (Parameters: matrix BLOSUM62, Gap cost 11 1, Max E-values 10^–10^) against a local curated fungal protein database (updated on January 2021, available at http://ciencias.uchile.cl/investigacion/nuestros-centros/centro-de-biotecnologia). The annotations were transferred from blast-hits to query sequences and the annotations including% of transferred similarity, calculated mol wt, and length of blast-hit and exported as comma separated values. The molecular weight of query sequences was calculated as the product of residues lengths multiplied by 110 Da as average molecular weight of amino acids. The data was processed in Microsoft excel 16.56 and hit with similarity ≥ 40%, coverage ≥ 50%, E values ≤ 10^–10^, and the ratio of calculated molecular weight between query and blast-hit in the range of 0.7–1.3 were filtered. The filtered sequence list was used to extract sequences by name in Geneious Prime. These annotated ORFs were classified according to cellular function predicted using the KAAS—KEGG Automatic Annotation Server (Moriya et al., 2007), using default parameters and gene data set for Eukaryotes. The ORF expression levels were calculated as reads per kilobase per million (RPKM) mapped reads, as previously described (Mortazavi et al., 2008), and ORFs with RPKM ≥ 10 in any of the conditions were considered for further analysis. The percentage of amino acids classified according to their flexibility index (Li et al., 2006; Rao et al., 2011), as very flexible (E, G, K, N, Q, and S) and moderately flexible (A, D, H, I, P, R, T, and V), were calculated for each annotated ORF. Furthermore, the flexibility of each translated ORF was calculated in three classes of predictions from rigid to flexible (0, 1, and 2) using MEDUSA, a deep learning-based protein flexibility tool that uses information from homologous protein sequences and amino acid physicochemical properties as input to assign a flexibility class to each protein sequence position using default parameters (Vander Meersche et al., 2021). The percentages of amino acids grouped as very flexible (Vf), very flexible plus moderately flexible (VMf), and the more flexible calculated by MEDUSA, class 2 (M2), and class 1 plus class 2 (M1 + 2), were compared among yeasts and ORFs grouped by cellular function applying one-way ANOVA followed by a *post hoc* Tukey analysis. The data distribution was visualized using histograms and quantile-quantile (QQ) plots and did not find any indication that the data did not have a normal distribution. Those parameters in which significant differences were found (significance threshold of 0.05) were considered to estimate their probable correlation to yeast growth parameters. The parameter mean differences were plotted vs. the corresponding differences among yeasts in their optimal temperature for growth (OTG) or growth rate (GR) determined in previous work (Baeza et al., 2021), and linear regressions were applied, considering only plots having at least three points.

### Protein Modeling and Structural Analysis

Enzyme 3D models were built by homology modeling using SWISS-MODEL^1^ (Guex et al., 2009; Bertoni et al., 2017; Bienert et al., 2017; Waterhouse et al., 2018; Studer et al., 2020). Models with QMEAN >–4.0 were selected and further checked using Verify3D (Bowie et al., 1991; Lüthy et al., 1992). Models were visualized and analyzed using UCSF Chimera software (Pettersen et al., 2004). The volume of the active site of the studied enzymes was predicted using the MOLEonline server^2^ (Pravda et al., 2018). The active site volume of templates was predicted by adjusting the probe radius and interior threshold settings for each individual model and used to predict the active site volume of the corresponding orthologous enzymes. The composition of amino acids of different protein structural levels was manually determined using categorization based on EMBOSS Pepstats software (Madeira et al., 2019): tiny (A, C, G, S, and T), small (A, C, D, G, N, P, S, T, and V), aliphatic (A, I, L, and V), aromatic (F, H, W, and Y), non-polar (A, C, F, G, I, L, M, P, V, W, and Y), polar (D, E, H, K, N, Q, R, S, and T), charged (D, E, H, K, and R), basic (H, K, and R), and acidic (D and E). Furthermore, the contents of hydrogen bonds and apolar solvent-excluded surfaces were determined. The ionic interactions were predicted using PIC software (Tina et al., 2007).

## Results

### Transcriptomes, Gene Identification, and Expression Analysis

The number of contigs assembled with length ≥210 nt ranged from 17,877 for *C. sake* incubated at 4°C to 34,096 for *Tetracladium* sp. incubated at 22°C. The total ORFs ≥ 210 nt predicted ranged from 19,896 for *Tetracladium* sp. incubated at 30°C to 99,705°C for *L. creatinivorum* incubated at 22°C (Supplementary Table 1). After filtering according to the methods mentioned in the Materials and Methods considering Blastp results, cellular function prediction, and RPKM values, the annotated ORFs ranged from 802 to 1,584, hereafter called putative genes. The yeasts with a higher number of putative genes were *W. anomalus*, *Tetracladium* sp., and *V. victoriae*, and those with lower numbers were *Cryptococcus* sp. and *P. glacialis*. The putative genes were classified according to predicted cellular function at three levels, from more general to specific (Supplementary Table 2). At the middle level, the pathways in which more putative genes were classified were carbohydrate metabolism, amino acid metabolism, and translation. The top five pathways were ribosome, oxidative phosphorylation, spliceosome, pyruvate metabolism, and endocytosis at the more specific level. There were pathways found only in one yeast: the apelin signaling pathway and drug metabolism enzymes in *C. sake*, D-glutamine and D-glutamate metabolism and flavone and flavonol biosynthesis in *V. victoriae*, and glycosaminoglycan biosynthesis chondroitin sulfate/dermatan sulfate, lipopolysaccharide biosynthesis, steroid degradation, polyketide sugar unit biosynthesis, and cell adhesion molecules in *Tetracladium* sp. The pathways found only in two or three yeasts were other glycan degradation, glycosaminoglycan degradation, cutin suberine and wax biosynthesis, carotenoid biosynthesis, phenazine biosynthesis, biosynthesis of ansamycins, glycosaminoglycan biosynthesis-heparan sulfate/heparin, and aflatoxin biosynthesis.

**FIGURE 1:**
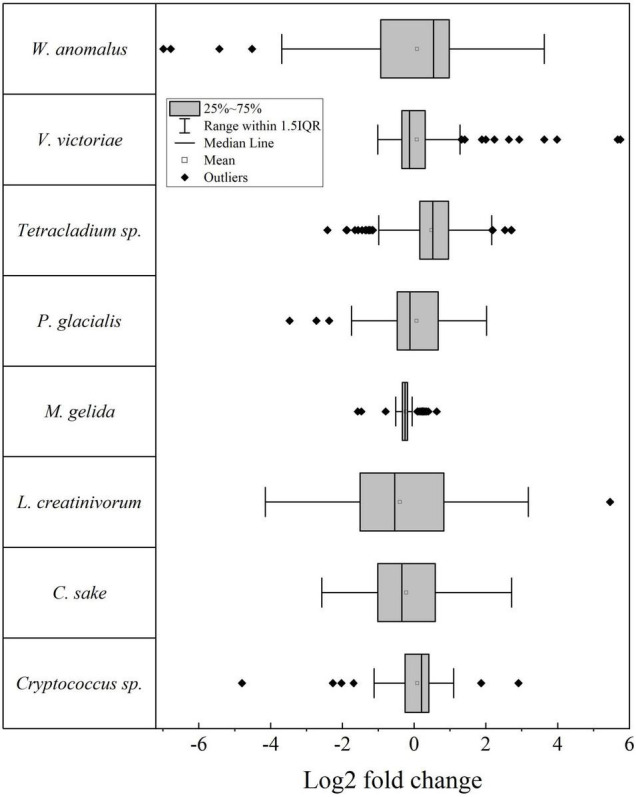
Box plot for the log_2_ fold change of putative genes between yeasts cultivated at high temperature and 4°C. IQR, Interquartile range.

**FIGURE 2:**
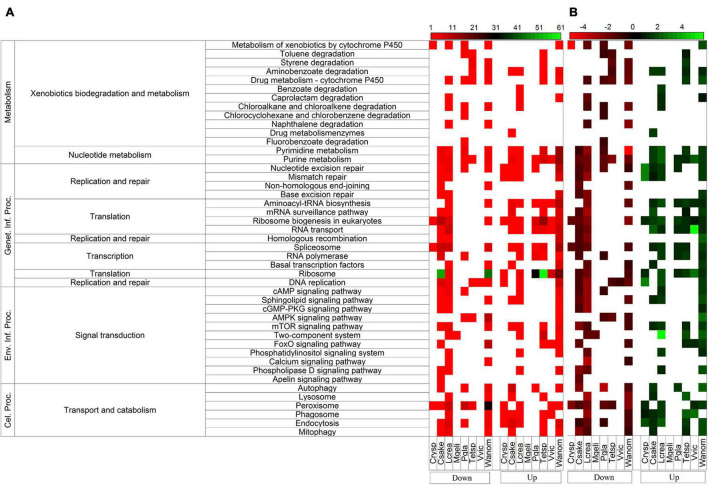
Differentially expressed putative genes classified by metabolic pathways between yeasts cultivated at high temperature (HT) and 4°C. The number of differentially expressed genes (DEGs) is shown in panel **(A)**, and the average of Log_2_-fold change (log_2_FC) by pathway is shown in panel **(B)**. Down, downregulated DEGs; Up, upregulated DEGs. Genet. Inf. Proc, Genetic Information Processing; Env. Inf. Proc, Environmental Information Processing; Cel. Proc., Cellular Processes.

The differential gene expression was determined for putative genes for each yeast when cultivated at high temperature vs. 4°C and expressed as log_2_-fold change (log_2_FC), the results shown in Figure 1 (*p* ≤ 0.05). The number of putative genes with positive log_2_FC values was higher than the number of genes with negative log_2_FC values in four yeasts (highest in *Tetracladium* sp.) and lower in four yeasts (lowest in *M. gelida*). Considering only significant differentially expressed putative genes (| log_2_FC| ≥ 1), the highest number of differentially expressed genes (DEGs) was found in *W. anomalus*, followed by *Tetracladium* sp. and *C. sake*, and lower numbers were found in *M. gelida* and *Cryptococcus* sp. Most yeasts had more upregulated than downregulated DEGs, except for *L. creatinivorum* and *C. sake*. Instead, there were only downregulated DEGs in *M. gelida*. Concerning general cellular pathways, a higher number of DEGs was found for genetic information processing, highest in *W. anomalus* and *Tetracladium* sp. At the middle level were translation and transport and catabolism, and at specific level were ribosome, peroxisome, and ribosome biogenesis in eukaryotes (Figure 2A). Considering the average of DEGs by metabolic pathway (Figure 2B), at the middle level, lower values were found for xenobiotic biodegradation and metabolism in *W. anomalus* and replication and repair and translation in *C. sake*. Higher values were found for replication and repair in *W. anomalus* and translation in *Tetracladium* sp. and *P. glacialis*. At the specific pathway level, lower averages were found for naphthalene degradation and non-homologous end-joining in *W. anomalus* and the cGMP-PKG signaling pathway in *L. creatinivorum*, while higher values were found for phospholipase D signaling and homologous recombination in *W. anomalus* and benzoate degradation in *L. creatinivorum*.

The gene expression changes between HT and 4°C were quite variable among yeasts. For example, the pathways downregulated, on average, at the general level were metabolism, cellular processes, and genetic information processing in 4, 3, and 2 yeasts, respectively. At the middle level were nucleotide metabolism, replication and repair, and translation in 3, 5, and 3 yeasts, respectively, and at the specific level were aminoacyl-tRNA biosynthesis, peroxisome, nucleotide excision repair, and autophagy in 1, 6, 3, and 1 yeasts, respectively. Statistical comparison analyses were performed to determine how differently the gene expression changed among yeasts, considering the DEGs at the different levels of cellular pathways. At the general level, higher differences among yeasts were found for metabolism and genetic information processing. *L. creatinivorum* and *C. sake* differed in cellular processes, and no differences were found for the environmental information processing pathway. At the middle level, higher differences were found for translation and carbohydrate metabolism, while at the specific level, the higher differences were found for ribosome and ribosome biogenesis in eukaryotes (Supplementary Figure 1A). The yeasts that displayed more differences from other yeasts were *C. sake*, *L. creatinivorum*, and *V. victoriae*. Higher differences between yeast pairs were observed for *C. sake* vs. *P. glacialis* and *Tetracladium* sp., *L. creatinivorum* vs. *V. victoriae* and *W. anomalus*, and *V. victoriae* vs. *W. anomalus*. When yeasts were clustered according to their differences (Supplementary Figure 1B), the yeasts *M. gelida* and *Cryptococcus* sp. appear close in a group with *C. sake*, *L. creatinivorum*, and *W. anomalus*. In another group, *P. glacialis* appeared close to *Tetracladium* sp., together with *V. victoriae*.

**FIGURE 3:**
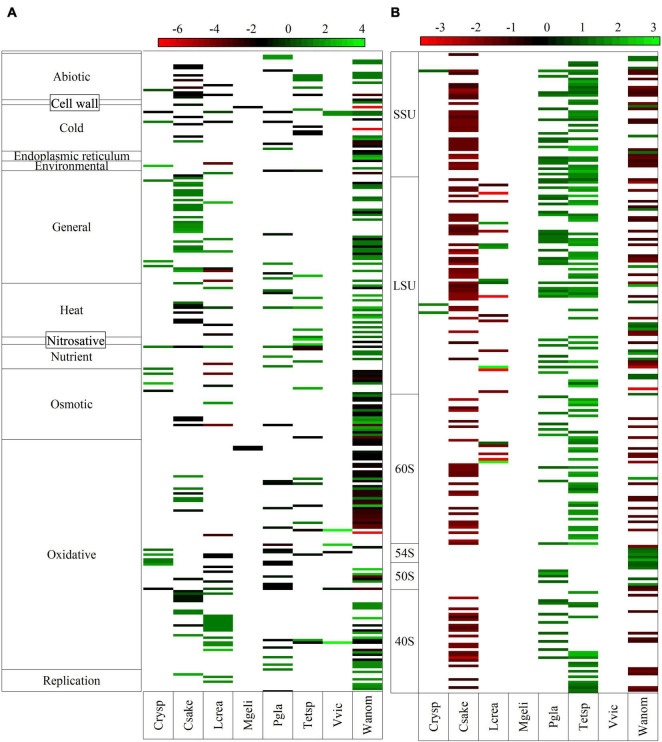
Log_2_-fold change of putative genes classified in different kinds of stress responses **(A)** and ribosomal subunits **(B)**.

An interesting aspect is the expression changes of genes encoding ribosomal subunits and described to function in different stress responses. DEGs related to responses to several kinds of stress, up- and downregulated, were found in all yeasts, with higher numbers belonging to oxidative, general, osmotic, heat, and cold conditions (Figure 3A and Supplementary Table 3). A higher number of stress-related DEGs was found by far in *W. anomalus*, followed by *C. sake* and *L. creatinivorum*, while a lower number was found in *V. victoriae* and *M. gelida*.

The number of upregulated DEGs involved in general stress was markedly higher than the number of downregulated DEGs, especially in *W. anomalus* and *C. sake*. In the case of oxidative and cold stress, the associated downregulated DEGs were higher than the upregulated DEGs, markedly in *W. anomalus* and *P. glacialis*. In terms of protein identity, the enzymes alcohol dehydrogenase appeared downregulated in five yeasts, and upregulated in *V. victoriae*. The 20S proteasome subunit, 26S proteasome subunit, chaperone, and osmolarity two component system were upregulated in four yeasts. A high number of DEGs identified as proteasome subunits were found in *C. sake* (22 in total, all upregulated) and *W. anomalus* (17 in total, 16 upregulated). In the case of putative cytochrome C oxidase subunits, the majority found in *C. sake*, *W. anomalus*, *P. glacialis*, and *M. gelida* were downregulated, whereas in *L. creatinivorum*, they were upregulated. In the case of DEGs identified as ribosomal subunits, higher numbers were found in *C. sake*, *Tetracladium* sp., and *W. anomalus*, only 3 in *Cryptococcus* sp., and none in *V. victoriae* and *M. gelida* (Figure 3B). In *C. sake* all putative ribosomal subunit genes found were downregulated, and in *W. anomalus* and *L. creatinivorum*, 70 and 62%, respectively, were downregulated. Contrary results were observed in *P. glacialis* and *Tetracladium* sp., in which all ribosomal subunits found were upregulated.

### Comparative Analysis of Flexibility Based on Amino Acid Composition

**FIGURE 4:**
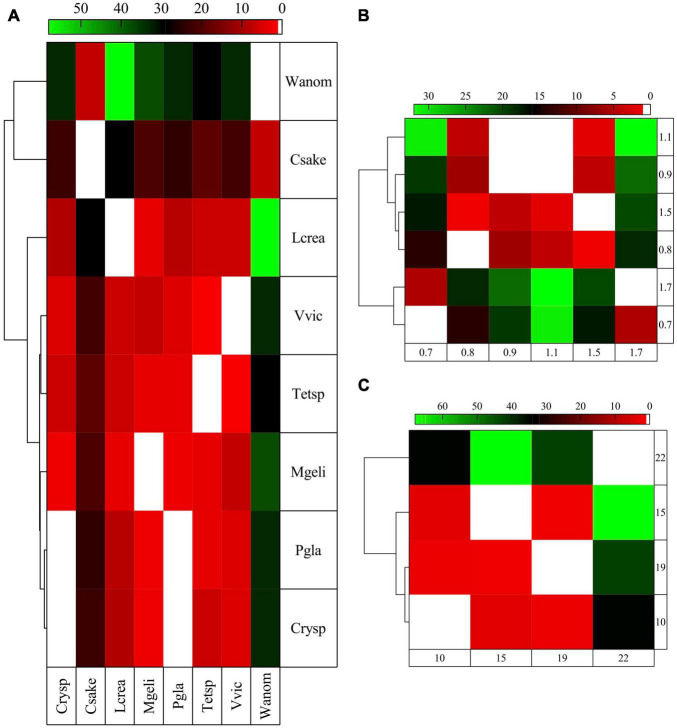
Comparisons among yeasts of the flexibility of translated proteins classified in cellular pathways. The flexibility was estimated as percentages of very flexible (Vf) and very flexible plus moderately flexible (VMf) amino acids and M2 and M1 + 2 levels, calculated by MEDUSA. The proteins grouped in cellular pathways were compared, and those with significant differences (Tukey *post hoc* tests, *p* < 0.05) are shown. The comparisons were performed among all yeasts individually **(A)** and grouped according to their growth rates **(B)** (h-1) and their optimal temperature for growth **(C)** (°C).

Protein flexibility was estimated from translated putative genes as percentages of Vf, VMf, M2, and M1 + 2 and compared among yeasts considering all genes and classified by cellular pathways. At the global level, the yeasts *W. anomalus* and *C. sake* displayed the highest differences compared with the other yeasts and were clustered with *Cryptococcus* sp., *P. glacialis*, and *M. gelida* (Supplementary Figure 2A). The yeasts *L. creatinivorum*, *Tetracladium* sp., and *V. victoriae* conformed to another cluster, in which the last two yeasts were closer. When the comparison was performed for yeasts grouped according to their Gr, those having higher and lower Gr were mixed clustered (Supplementary Figure 2B). In the case of OTGs, the yeasts having OTGs from 10 to 19°C were grouped, excluding those having OTGs of 22°C (Supplementary Figure 2C). When the comparisons were performed using the putative genes grouped by cellular pathways, the yeasts *C. sake*, *W. anomalus*, and *L. creatinivorum* displayed higher differences from the other yeasts. While *C. sake* and *W. anomalus* formed a cluster, *L. creatinivorum* was grouped with the other five yeasts, in which *P. glacialis* with *Cryptococcus* sp. and *V. victoriae* with *Tetracladium* sp. were closer (Figure 4A). In the clustering obtained from comparisons from yeasts grouped according to their Gr, yeasts with high and low Gr values were clustered together (Figure 4B) and grouped according their OTG. The yeasts with OTGs of 10 and 19°C were closer in a group with those having OTGs of 15°C, while yeast with OTGs of 22°C were apart (Figure 4C).

A consistency between growth parameters and how yeasts were clustered was observed concerning yeast OTG. The probable correlation between the calculated flexibility of translated proteins and yeast growth parameters was analyzed using the data from comparisons with significant differences (results in Supplementary Table 4). Considering results having R^2^ ≥ 0.7, at the global level, an inverse correlation between% of M2 and OTG was found in the comparison of all yeasts. When yeasts were compared grouped according to growth parameters, there were direct correlations between VMf and Gr in yeasts with low OTG and between Vf and OTG in yeasts with low Gr. When comparisons were performed with translated proteins grouped according to cellular pathways, most correlations were for Vf and generally direct. When all yeasts were compared, inverse correlations were found between M2 and Gr in replication and repair, M1 + 2 and Gr in genetic information processing, and VMf and OTG in lipid metabolism. Direct correlations were found between Vf and Gr in translation and transcription and VMf and glycan biosynthesis and metabolism. Furthermore, direct correlations were found between Vf and OTG in amino acid metabolism, biosynthesis of amino acids, genetic information processing, metabolism of cofactors and vitamins, translation, microbial metabolism in diverse environments, energy metabolism, biosynthesis of secondary metabolites, cell cycle, meiosis, spliceosome, and cell growth and death. In comparing yeasts with high OTG, an inverse correlation between M2 and Gr in general metabolism was found. In yeasts with low Gr, direct correlations were found between Vf and OTG in the cell cycle, cell growth and death, cellular processes, genetic information processing, translation, biosynthesis of cofactors, metabolism, and carbohydrate metabolism.

### Protein Structural Properties and Differential Expression

As mentioned above, there were differences among yeasts in DEGs associated with stresses and ribosomal subunits. The predicted flexibility of ribosomal and proteasome subunits and putative genes encoding stress responses, grouped into up- and downregulated genes, were compared finding significant differences primarily for stress-associated genes (Supplementary Figure 3). However, significant differences were found not exclusively in up- vs. downregulated DEGs but also in up- vs. upregulated and downregulated vs. downregulated DEGs. In this way, at least for the evaluated DEGs, there was no correlation between their expression change and global flexibility.

Forty-nine putative isozymes displayed up- and downregulation in yeast and among yeasts, which were characterized concerning structural properties described as necessary in cold adaptation. Regarding the global calculated flexibility, there were no significant differences among up- vs. downregulated putative isozymes. For more structural analysis, the 3D models were successfully predicted for 17 putative isozymes and used to calculate parameters such as amino acid interactions, apolar solvent excluded surface, secondary structures, and volume and flexibility of catalytic sites. No significant differences were found between putative up- and downregulated isozymes in any of the structural properties compared, and the distribution of parameter values was similar in up- and downregulated isozymes (Supplementary Figure 4). Next, the comparison was performed among the isozymes from Antarctic yeasts and the orthologs from mesophiles used as templates for 3D structure prediction. As shown in Supplementary Table 5, significant differences were found for the percentages of amino acids in α-helices and β-sheets, charged amino acids in β-sheets, H- bonds, ionic interactions, and amino acids in coil structure, which were, except the last one, higher in mesophilic than Antarctic yeasts. Furthermore, the analysis was performed for predicted active sites regarding their flexibility and volume, finding no significant differences between the Antarctic vs. mesophilic. As shown in Supplementary Figure 5, the values were higher or lower for the Antarctic or mesophilic isozymes analyzed, being generally higher in the mesophilic isozymes.

### Analysis of Codon Usage Bias

**FIGURE 5:**
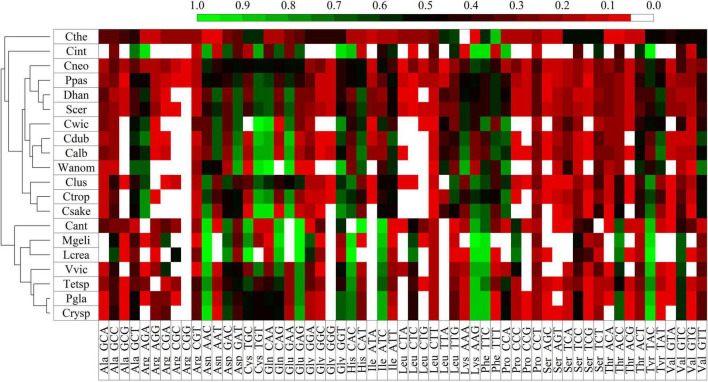
Clustering of yeasts according to codon frequencies. The codon frequencies were calculated using highly expressed ORFs (≥1,000) in the eight Antarctic yeasts studied here, from the Kazusa coding usage database and from (Baeza et al., 2015). Scer, *S. cerevisiae*; Ppas, *Pichia pastoris*; Cant, *Candida antarctica*; Cthe, *Candida thermophila*; Calb, *Candida albicans*; Cneof, *Cryptococcus neoformans* var. Grubii; Cwic, *Candida wickerhamii*; Cdub, *Candida dubliniensis*; Cint, *Candida intermedia*; Ctro, *Candida tropicalis*; Clusi, *Clavispora lusitania*; DhanCBS767, *Debaryomyces hansenii* CBS767; Huva, *Hanseniaspora uvarum*; Prhod, *Phaffia rhodozyma*; Mfur, *Malassezia furfur*; Mpach, *Malassezia pachydermatis*; Cglab, *Candida glabrata*; Ptsuk, *Pseudozyma tsukubaensis*; Cposa, *Coccidioides posadasii*; KlacNRRL Y-1141, *Kluyveromyces lactis* NRRL Y-1141; Xd385, *Xanthophyllomyces dendrorhous* UCD67-385.

Codon usage bias (CUB) was analyzed in each yeast according to the expression levels of their putative genes, which were classified into six groups considering their RPKM values: 1–50, 51–100, 101–300, 301–500, 501–1,000, and >1,000. A total of 22% of ORFs showed variation in their classification between yeasts cultivated at HT and 4°C. Therefore, the classification was also performed considering the maximum RPKM value (M) between both conditions. The codon preference was calculated for each group and compared in and among yeasts, finding higher variations in comparison performed with ORFs grouped according to their M values. In the comparison among groups, the top five amino acids with significant CUB variations in yeast were Thr, Gly, Leu, Ser, and Ala, while among yeasts, Leu, Ser, Arg, Gly, and Pro. There was a direct relationship in the differences in expression level and codon usage between groups in and among yeasts (Supplementary Figure 6). Furthermore, the groups with low expression levels showed high differences among them, contrary to the highly expressed groups. For more CUB analysis, the ORFs with RPKM > 1,000 grouped according to their M values were considered. The codon frequencies were compared among yeasts, and the codons with higher significant variation corresponded to the amino acids Ser, Leu, Arg, Gly, and Thr. Supplementary Figure 7 shows the clustering of yeasts according to their significant differences in codon frequencies, in which two groups can be observed, one conforming to *W. anomalus* and *C. sake*. In the group conforming to the other six yeasts, there were two subgroups, one containing *M. gelida* and *L. creatinivorum* and the other containing *Tetracladium* sp. close to *V. victoriae*, and *Cryptococcus* sp. close to *P. glacialis*. As observed in Table 1, some codons are commonly preferred in most yeasts, such as GCC and GCT for Ala, AAC for Asn, ATC for Ile, GAG for Glu, CTC for Leu, TTC for Phe, TCC for Ser, ACC for Thr, TAC for Tyr and GTC for Val. If yeasts are compared regarding their codon usage frequencies, they appear grouped, as shown in Supplementary Figure 7. Considering the yeast growth parameters, *W. anomalus* and *C. sake* were the yeasts with the highest OTG (22°C) but different growth rates (0.7 and 1.7 h^–1^, respectively) and appeared in a group separated from the other yeasts. The clustering of the six yeasts with OTGs from 10 to 19°C did not appear to be related to either OTG or growth rate. The analysis was expanded by incorporating codon frequencies from other yeasts available in the Kazusa codon usage database. As shown in Figure 5, Antarctic yeasts with OTGs equal to or less than 19°C appeared in a group including the yeast *Candida antarctica*. Instead, the yeasts *C. sake* and *W. anomalus* appeared grouped with mesophilic yeasts, *C. sake* with *Candida tropicalis* and *Clavispora lusitaniae*, and *W. anomalus* with *Candida dubliniensis*, *Candida albicans*, and *Candida wickerhamii*. There were marked codon preferences in the group conforming to the Antarctic yeasts studied here and *C. antarctica* that was not observed in other yeasts: AAC for Asn, GAG for Glu, CAC for His, ATC for Ile, AAG for Lys, TTC for Phe, AAC for Thr, TAC for Tyr.

**TABLE 1:** Relative synonymous codon usage in cold-adapted yeasts.

AA	Cod	Crysp	Csake	Lcrea	Mgeli	Pgla	Tetsp	Vvic	Wanom
Ala	GCA	0.09	0.11	0.05	0.04	0.09	0.22	0.13	0.15
	GCC	0.41	0.37	0.62	0.53	0.39	0.37	0.44	0.15
	GCG	0.05	0.02	0.06	0.05	0.07	0.07	0.03	0.01
	GCT	0.45	0.50	0.28	0.38	0.45	0.34	0.39	0.70
Arg	AGA	0.04	0.78	0.01	0.06	0.05	0.26	0.05	0.68
	AGG	0.30	0.03	0.09	0.23	0.30	0.03	0.22	0.03
	CGA	0.30	0.01	0.04	0.62	0.28	0.14	0.59	0.01
	CGC	0.04	0.00	0.54	0.02	0.05	0.21	0.01	0.02
	CGG	0.01	0.02	0.02	0.01	0.02	0.02	0.02	0.00
	CGT	0.30	0.13	0.30	0.07	0.30	0.35	0.09	0.25
Asn	AAC	0.90	0.78	0.98	0.95	0.89	0.80	0.87	0.51
	AAT	0.08	0.21	0.02	0.02	0.10	0.20	0.11	0.48
Asp	GAC	0.63	0.47	0.76	0.66	0.59	0.46	0.55	0.17
	GAT	0.36	0.53	0.24	0.34	0.40	0.53	0.46	0.83
Cys	TGC	0.55	0.08	0.99	0.77	0.51	0.62	0.28	0.08
	TGT	0.45	0.92	0.01	0.23	0.49	0.38	0.72	0.92
Gln	CAA	0.49	0.91	0.12	0.10	0.51	0.41	0.29	0.97
	CAG	0.51	0.09	0.88	0.90	0.49	0.59	0.71	0.03
Glu	GAA	0.17	0.87	0.04	0.05	0.21	0.21	0.24	0.92
	GAG	0.83	0.12	0.96	0.95	0.80	0.79	0.76	0.08
Gly	GGA	0.18	0.16	0.57	0.67	0.19	0.30	0.35	0.07
	GGC	0.11	0.05	0.22	0.06	0.11	0.24	0.09	0.04
	GGG	0.02	0.04	0.02	0.03	0.04	0.02	0.02	0.04
	GGT	0.69	0.75	0.18	0.24	0.66	0.44	0.54	0.85
His	CAC	0.73	0.66	0.96	0.91	0.70	0.77	0.82	0.29
	CAT	0.25	0.30	0.04	0.07	0.28	0.21	0.16	0.69
Ile	ATA	0.01	0.04	0.01	0.01	0.02	0.02	0.02	0.05
	ATC	0.85	0.46	0.83	0.90	0.81	0.69	0.72	0.39
	ATT	0.14	0.49	0.17	0.10	0.17	0.29	0.24	0.57
Leu	CTA	0.02	0.00	0.01	0.01	0.02	0.05	0.17	0.05
	CTC	0.43	0.04	0.71	0.61	0.42	0.40	0.41	0.02
	CTG	0.03	0.01	0.05	0.05	0.04	0.07	0.02	0.01
	CTT	0.19	0.07	0.16	0.20	0.19	0.19	0.16	0.07
	TTA	0.01	0.38	0.01	0.00	0.02	0.03	0.02	0.59
	TTG	0.31	0.50	0.06	0.13	0.31	0.26	0.22	0.27
Lys	AAA	0.11	0.28	0.06	0.04	0.15	0.13	0.16	0.81
	AAG	0.89	0.72	0.94	0.96	0.85	0.87	0.84	0.19
Phe	TTC	0.87	0.67	0.92	0.91	0.86	0.75	0.84	0.59
	TTT	0.07	0.33	0.08	0.04	0.08	0.25	0.13	0.39
Pro	CCA	0.52	0.68	0.03	0.04	0.49	0.55	0.20	0.78
	CCC	0.19	0.04	0.72	0.65	0.19	0.17	0.51	0.01
	CCG	0.06	0.02	0.04	0.02	0.08	0.05	0.02	0.00
	CCT	0.23	0.26	0.18	0.27	0.23	0.22	0.28	0.21
Ser	AGC	0.08	0.03	0.07	0.03	0.08	0.15	0.05	0.03
	AGT	0.05	0.08	0.01	0.01	0.05	0.07	0.05	0.11
	TCA	0.10	0.11	0.02	0.02	0.13	0.21	0.11	0.43
	TCC	0.49	0.29	0.41	0.53	0.47	0.30	0.39	0.10
	TCG	0.07	0.06	0.38	0.20	0.08	0.09	0.10	0.01
	TCT	0.21	0.42	0.11	0.20	0.20	0.18	0.30	0.33
Thr	ACA	0.04	0.10	0.03	0.02	0.05	0.17	0.08	0.17
	ACC	0.68	0.46	0.72	0.77	0.65	0.46	0.57	0.23
	ACG	0.05	0.03	0.08	0.03	0.05	0.10	0.03	0.01
	ACT	0.23	0.42	0.18	0.18	0.24	0.28	0.32	0.58
Tyr	TAC	0.91	0.73	0.98	0.94	0.89	0.78	0.90	0.44
	TAT	0.07	0.24	0.02	0.01	0.09	0.18	0.10	0.55
Val	GTA	0.05	0.07	0.02	0.01	0.07	0.05	0.06	0.03
	GTC	0.59	0.36	0.69	0.67	0.55	0.60	0.61	0.20
	GTG	0.05	0.07	0.06	0.05	0.07	0.11	0.03	0.06
	GTT	0.31	0.50	0.24	0.27	0.31	0.24	0.31	0.72

*The codon frequencies were calculated using highly expressed putative genes (≥1,000). The codons with clear preference in each amino acid are highlighted in light gray.*

## Discussion

The ORFeomes of eight Antarctic yeasts were assembled when cultivated in the optimal temperature range and switched to 4°C. Strict terms were used to predict putative genes, and higher numbers were found in *W. anomalus*, *Tetracladium* sp., and *V. victoriae*. According to predicted cellular functions, a high number of putative genes were classified in carbohydrate metabolism, amino acid metabolism, and translation. The top five pathways were ribosome, oxidative phosphorylation, spliceosome, pyruvate metabolism, and endocytosis at the more specific level. In predicting the cellular functions of putative genes, 159 specific pathways were found, of which 121 were found in eight yeasts and 136 in at least six yeasts. Other pathways were found only in 1–3 yeasts, such as biosynthesis of other secondary metabolites, glycan biosynthesis and metabolism lipid metabolism, metabolism of other amino acids, metabolism of terpenoids and polyketides, signal transduction, signaling molecules and interaction, and xenobiotic biodegradation and metabolism, similar to results from the analysis of draft genomes (Baeza et al., 2021).

Putative genes associated with the response to all main kinds of stress were identified, and those differentially expressed belonged mostly to oxidative, general, and osmotic stress responses, and putative genes up- and downregulated were found (Table 2). Many downregulated genes associated with stress were observed in the yeasts *W. anomalus* and *C. sake*. In general, a relatively low number of upregulated heat-shock proteins, chaperones that play roles in routine biological processes and responses to stresses (Tiwari et al., 2015), were found in *C. sake*, *Tetracladium* sp., and *W. anomalus* but not in *Cryptococcus* sp., *V. victoriae*, and *M. gelida*. Other canonical cold stress proteins are fatty acid desaturases (Cossins et al., 2002; Garba et al., 2017), which were found to be upregulated in *Tetracladium* sp. and in *W. anomalus*, downregulated in *C. sake* and not in the rest of the yeasts. An interpretation of these results could be that the proteins and lipid membranes are adapted to cold in these yeasts and were not significantly affected by the temperature switch to 4°C. Therefore, a significant expression of genes encoding for canonical chaperones or fatty acid desaturases described for cold stress in mesophylls would no needed in these cold adapted yeasts. Alcohol dehydrogenase enzymes were upregulated in V. victoriae like described in another Antarctic yeast, *Rhodotorula frigidialcoholis*, when exposed to temperature change from 23 to 0°C (Touchette et al., 2021). The authors suggested a metabolic change in *R. frigidialcoholis* that increased xylitol production and redirected pentose phosphate pathway molecules to ethanol fermentation, which would benefit its survival at low temperatures. The yeast *L. creatinivorum* would be responding to oxidative stress because of the upregulation of some subunits of mitochondrial cytochrome c oxidase found. This protein complex is the primary site of cellular oxygen consumption, essential for aerobic energy generation, and roles in adaptation to stress conditions such as hypoxia, oxidative or low glucose levels (Khalimonchuk and Rödel, 2005; Timón-Gómez et al., 2020). Nevertheless, genes encoding for alcohol dehydrogenase enzymes and cytochrome c oxidase subunits were downregulated in other yeasts, raising the different ways that yeasts were affected and responded to cold, as commented below.

**TABLE 2:** Summary of differential expressed genes in Antarctic yeasts.

	TC (°C)	Total		Stress			Proteasome subunits		Ribosomal subunits	
			
		Down	Up	Gen	Osm	Oxi	Down	Up	Down	Up
*C. sake*	18	174	188	24	3	16		22	105	
*Cryptococcus sp.*	6	39	48	3	3	6		3		3
*L. creatinivorum*	18	245	187	7	4	19	3	2	13	8
*M. gelida*	6	8				2				0
*P. glacialis*	11	111	69	4	1	18				63
*Tetracladium sp.*	18	110	170	1	2	8				113
*V. victoriae*	11	12	75			5				
*W. anomalus*	26	503	534	23	25	56	1	16	69	30

Putative genes encoding proteasomal subunits were considerably upregulated in *C. sake* and *W. anomalus*, while a high number of genes encoding ribosomal subunits were downregulated (Table 2). In contrast, minor expression changes in genes for proteasome and ribosome subunits were found in *Cryptococcus* sp., *V. victoriae*, and *M. gelida*. A considerable number of genes for ribosomal subunits were upregulated in *P. glacialis* and *Tetracladium* sp. Based on these findings and that proteosomes are complex and involved in proteolytic pathways, including normal and damaged proteins (Goldberg, 2003; Wang et al., 2010) and ribosome biogenesis is considered one of the most energy-demanding cellular processes (Shore et al., 2021), clearly *C. sake* and *W. anomalus* were the yeasts most affected by the temperature switch to 4°C. Therefore, we could speculate that their response is rather slow down cellular processes and prepare to enter dormancy. In *Shewanella oneidensis*, the downregulation of genes related to amino acid biosynthesis, protein synthesis, and protein fate after cold shock was proposed as a mechanism of downregulation of bacterial metabolism (Gao et al., 2006; De Maayer et al., 2014). The yeasts *P. glacialis* and *Tetracladium* sp. have intermediate OTGs, and the upregulation of ribosomal subunits observed could be an effort to maintain their growth at lower temperatures, which may not be necessary for *Cryptococcus* sp., *V. victoriae*, and *M. gelida*, yeast with lower OTGs.

Beyond stress-related genes, the total number of differentially expressed genes was directly related to the temperature variation (ΔT) that each yeast faced (Table 2). The highest number of overexpressed and repressed putative genes was found in *W. anomalus*, the yeasts that experienced the highest ΔT (26°C), followed by yeasts with a ΔT of 18°C. The lowest number of DEGs was found in *Cryptococcus* sp. and *M. gelida* (ΔT = 6°C), and in the last one, only downregulated DEGs were found, possibly an indication of the differences in adaptive mechanisms developed in this group of cold-adapted yeasts inhabiting the same terrestrial habitat from Antarctica. Yeast with lower OTG would have adaptations to grow well or adapt their metabolism to the applied cold conditions. Instead, yeasts with higher OTGs would have to make significant adjustments to tolerate lower temperatures.

In the clustering of yeasts either according to gene expression changes or estimated protein flexibility globally and by cellular pathways, the results were similar, with *C. sake* and *W. anomalus* grouped and separated from the rest of the yeasts. Commonly *Cryptococcus* sp. was close to *M. gelida*, and the clustering of the other yeasts was more variable depending on the parameter considered. *C. sake* and *W. anomalus* have the same OTG, but *C. sake* grows faster, and *Cryptococcus* sp. has an OTG and Gr higher than *M. gelida* but lower than *C. sake* and *W. anomalus*. The rest of the yeasts have medium growth parameters. Therefore, it is not evident to establish a relationship between the clustering of yeasts and their growth parameters, except for extreme yeasts in which their differentiation may be more associated with their OTG. In the comparative analysis at the global level of calculated protein flexibilities vs. growth parameters of yeasts, no significant correlation was found in the comparison among all yeasts. However, a direct correlation was found between the content of moderately plus very flexible amino acids and Gr in yeasts having low OTG and between the content of very flexible amino acids and OTG in yeasts growing slowly. In comparisons by pathways, significant direct correlations were found for the content of very flexible amino acids: Gr in translation and transcription considering all yeasts; OTG for amino acid metabolism, biosynthesis of amino acids, genetic information processing, and metabolism of cofactors and vitamins considering all yeasts; and OTG for cell cycle, cell growth and death, cellular processes and genetic information in yeast grown slowly.

Several putative isozymes were found to be up- and downregulated in the same yeasts, mainly in *W. anomalus* or different yeasts, raising the possibility that the yeasts performed a replacement for enzymes more adequate to function at a lower temperature, as described for *Saccharomyces cerevisiae* when exposed to different stresses (Ansell et al., 1997; Postmus et al., 2012). Among the structural properties reported for cold-active enzymes are an increased number of small side-chain amino acids, reduced hydrophobicity in the core of the protein, an increased number and longer random coils, reduced number of ionic and hydrogen bond interactions and larger and more flexible active sites (Lonhienne et al., 2001; D’Amico et al., 2006; Jung et al., 2008; Feller, 2013; Santiago et al., 2016; Berg et al., 2019; Ang et al., 2021; Parvizpour et al., 2021; Yang et al., 2021; Yusof et al., 2021). When the up- vs. downregulated isozymes were compared in these properties, calculated from 3D structure models, no significant differences were found, but significant differences were found when compared to mesophyll counterparts used as a template for 3D modeling. Significant differences were fewer hydrogen bonds, ionic interactions, amino acids in α-helices and β-sheets, and an increased number of amino acids in random coils. These properties have been associated with a more flexible and less compact and stable protein structure (Lonhienne et al., 2001; D’Amico et al., 2006; Feller, 2013; Santiago et al., 2016; Ang et al., 2021; Yang et al., 2021). Predicted volume and flexibility of Antarctic enzymes’ active sites were higher or lower than its mesophilic counterpart depending on the enzyme compared, as reported in studies of draft genomes (Baeza et al., 2021). At the structural level, the adaptation of enzymes could be specific and variable for an enzyme, as stated for different enzyme families (Feller, 2013; Santiago et al., 2016).

The existence of specific codon usage bias at cold temperatures has been proposed from proteomic and genomic studies of cold-adapted microorganisms (Methé et al., 2005; Médigue et al., 2005; Su et al., 2016). To evaluate codon usage bias in the Antarctic yeasts studied here, codon usage bias was determined and compared among them. Independent of which codon was preferred, all yeasts displayed a strong codon preference in 11 amino acids: 7 in Asn, Cys, Gln and Thr, 6 in Asp, and only 3 in Leu and Ser. Although the codon preferences were variable among yeasts, there were preferred codons common in most, such as GCC and GCT for Ala, AAC for Asn, ATC for Ile, GAG for Glu, CTC for Leu, TTC for Phe, TCC for Ser, ACC for Thr, TAC for Tyr and GTC for Val. In the yeast *M. psychrophila*, which optimally grows at 12–15°C, codon usage bias was reported, especially GGA for Gly and CGA for Arg (Su et al., 2016), both coincident with results obtained here for *M. gelida*, and GGA for *V. victoriae*. Interestingly, when yeasts were clustered according to their codon usage preferences, *W. anomalus* and *C. sake* were grouped apart from the other yeasts, as determined based on gene expression and protein flexibility. Different clustering was obtained for the other Antarctic yeasts: *M. gelida* close to *L. creatinivorum*, *Tetracladium* sp. close to *V. victoriae*, and *Cryptococcus* sp. close to *P. glacialis*. When clustering was performed including other cold-adapted and mesophilic yeasts, Antarctic yeasts with OTGs equal to or less than 19°C appeared in a group including the yeast *Candida antarctica*. In this group, marked codon preferences were not observed in other yeasts: AAC for Asn, GAG for Glu, CAC for His, ATC for Ile, AAG for Lys, TTC for Phe, AAC for Thr, and TAC for Tyr. *C. sake* and *W. anomalus* appeared grouped with mesophilic yeasts, *C. sake* with *Candida tropicalis* and *Clavispora lusitaniae*, and *W. anomalus* with *Candida dubliniensis*, *Candida albicans*, and *Candida wickerhamii*. These results could support the proposition of the existence of a particular codon usage bias in cold-loving microorganisms, or at least in yeasts. The existence of codon usage bias is a topic of debate, and among the several proposed forces that determine codon usage bias is its coevolution with tRNA abundance that in turn influences the translation rate and protein folding (Higgs and Ran, 2008; Shah and Gilchrist, 2010; Angov, 2011; Novoa and Ribas de Pouplana, 2012; Trotta, 2013; Frumkin et al., 2018). The rate of protein synthesis is assumed to be lower at cold temperatures than at template temperatures, and the Antarctic yeasts with more similar codon usage bias were those with lower optimal growth temperatures. However, in recent work, preference for AT in the third base in psychrophile bacteria was reported (Duan and Guo, 2021), which was not found in the Antarctic yeasts studied here. Therefore, more supporting evidence is needed to raise the existence of cold-specific codon usage bias.

This study provided a global comparative analysis of the response to cold of different cold-adapted yeast species. Although the cold stress used in this work appears not extreme, it must be considered that the subAntarctic region has seasonal temperature changes, with maximal reported temperatures up to 12°C and even 20°C (Arnold et al., 2003; Carrasco et al., 2012). In this way, a temperature switch to 4°C can be considered an intermediate phase to lower temperatures that yeasts must sense to adapt their cellular functions. In general terms, yeasts with the lowest OTG displayed almost a half of changes at the transcriptomic level compared to those with the highest OTG, suggesting that these latter yeasts would decrease their metabolism to enter a dormant stage, which would be reversed if yeasts are submitted to upward temperature change, which is interesting to test.

## Data Availability Statement

The datasets presented in this study can be found in online repositories. The names of the repository/repositories and accession number(s) can be found below: NCBI database Bioprojects: *Cryptococcus* sp., PRJNA761919; *C. sake*, PRJNA761920; *L. creatinivorum*, PRJNA761921; *M. gelida*, PRJNA761923; *P. glacialis*, PRJNA761924; *Tetracladium* sp., PRJNA783217; *V. victoriae*, PRJNA761926; and *W. anomalus*, PRJNA761928, https://doi.org/10.34691/FK2/APMVNO.

## Author Contributions

SZ carried out the yeast cultures. VP and FG constructed and analyzed protein 3D models. SB and MB constructed and actualized the curated databases. SZ, VP, FG, and MB performed bioinformatic and statistical analyses. VC and JA contributed to the design of the experiments, discussion of the results, and manuscript writing. MB contributed the conceptualization of the study, writing the manuscript, project administration, and funding acquisition. All authors have read and agreed to the published version of the manuscript.

## Conflict of Interest

The authors declare that the research was conducted in the absence of any commercial or financial relationships that could be construed as a potential conflict of interest.

## Publisher’s Note

All claims expressed in this article are solely those of the authors and do not necessarily represent those of their affiliated organizations, or those of the publisher, the editors and the reviewers. Any product that may be evaluated in this article, or claim that may be made by its manufacturer, is not guaranteed or endorsed by the publisher.

## Footnotes

1
http://swissmodel.expasy.org/

2
https://mole.upol.cz/

## Supplementary Material

Supplementary Figure 1 Comparative analysis of gene expression changes among yeasts. The cellular pathways with significant differences (Tukey *post hoc* tests, *p* < 0.05) are shown. **(A)** Mean difference, yeast1 minus yeast2. **(B)** Clustering of yeasts according to the number of pathways in which they differ.

Supplementary Figure 2 Global comparisons of the flexibility of translated proteins among yeasts. The flexibility was estimated as percentages of very flexible (Vf) and very flexible plus moderately flexible (VMf) amino acids and class 2 (M2), and class 1 plus class 2 (M1 + 2) levels calculated by MEDUSA, compared among yeasts, and those with significant differences (Tukey *post hoc* tests, *p* < 0.05) are shown. The comparisons were performed among all yeasts individually **(A)** and grouped according to their growth rates **(B)** (h^–1^) and their optimal temperature for growth **(C)** (°C).

Supplementary Figure 3 Comparisons of the flexibility of proteins related to stress responses, proteasome subunits (Prot), and ribosomal subunits (Ribo). The proteins grouped as up- and downregulated were compared considering all and yeasts. The flexibility was estimated as percentages of very flexible (Vf) and very flexible plus moderately flexible (VMf) amino acids and class 2 (M2), and class 1 plus class 2 (M1 + 2) levels, calculated by MEDUSA. The mean difference of log_2_-fold change (Factor 1 minus 2) for comparisons with significant differences (Tukey *post hoc* tests, *p* < 0.05) are shown.

Supplementary Figure 4 Structural features of up- and downregulated isozymes. The structural features were calculated from 3D models constructed for each isozyme. **(A)** Percentages of flexibility and properties of amino acids. **(B)** Amino acid interactions, the fraction of apolar solvent excluding the surface (SES), and the fraction of amino acids by secondary structure. **(C)** catalytic site volume. **(D)** Percentage of amino acids having a property by secondary structures.

Supplementary Figure 5 Structural predicted active site in up- and downregulated isozymes. The structural features were calculated from 3D models constructed for each isozyme. The difference between Antarctic enzymes and mesophilic orthologs in flexibility predicted by MEDUSA **(A)** and the calculated volume of active site cavities **(B)** are shown.

Supplementary Figure 6 Codon frequency comparison among putative genes grouped according to their expression levels in **(A)** and among **(B)** yeasts. The ORFs were grouped according to their RPKM values in yeasts cultivated at high temperature (HT), 4°C, and the maximum value between conditions (M). The number of codons with significant differences between groups (Tukey *post hoc* tests, *p* < 0.05) is shown.

Supplementary Figure 7 Comparison of codon usage among yeasts considering the >1,000 RPKM group. The number of codons showing a significant difference (Tukey *post hoc* tests, *p* < 0.05) between yeast pairs is shown.

Supplementary Table 1 Summary of transcriptome sequencing results.

Supplementary Table 2 Number of ORFs classified according to metabolic pathways, using KAAS, KEGG Automatic Annotation Server, from more general (Path1) to more specific (Path3) pathway.

Supplementary Table 3 Log_2_ fold change for putative genes related to stress responses.

Supplementary Table 4 Correlation between amino acid parameters and growth parameters of yeasts. The parameters for which significant differences were found (Tukey *post hoc* tests, *p* < 0.05) were used for calculations. The comparisons were made considering the data from all yeast (All) or grouped according to growth parameters as those having high or low optimal temperature for growth (OTGH, OTGL) or high or low growth rate (GRH, GRL). Comparisons were performed globally **(A)** and according to the classification of ORFs by metabolic pathways **(B)**.

Supplementary Table 5 Structural comparisons between Antarctic and template orthologous.

## Abbreviations

OTG: optimal temperature for growth
GR: growth rate
HT: high temperature
RPKM: reads per kilo base per million mapped reads
DEGs: differentially expressed genes
Crysp: *Cryptococcus* sp.
Csake: *C. sake*
Lcrea: *L. creatinivorum*
Mgeli: *M. gelida*
Pgla: *P. glacialis*
Tetsp: *Tetracladium* sp.
Vvic: *V. victoriae*
Wanom: *W. anomalus*.
