# Supplementary figures and images for: Response to Cold: A Comparative Transcriptomic Analysis in Eight Cold-Adapted Yeasts
Source: Front Microbiol. 2022 Feb 23;13:828536. doi: 10.3389/fmicb.2022.828536 (PMC8905146; doi:10.3389/fmicb.2022.828536)

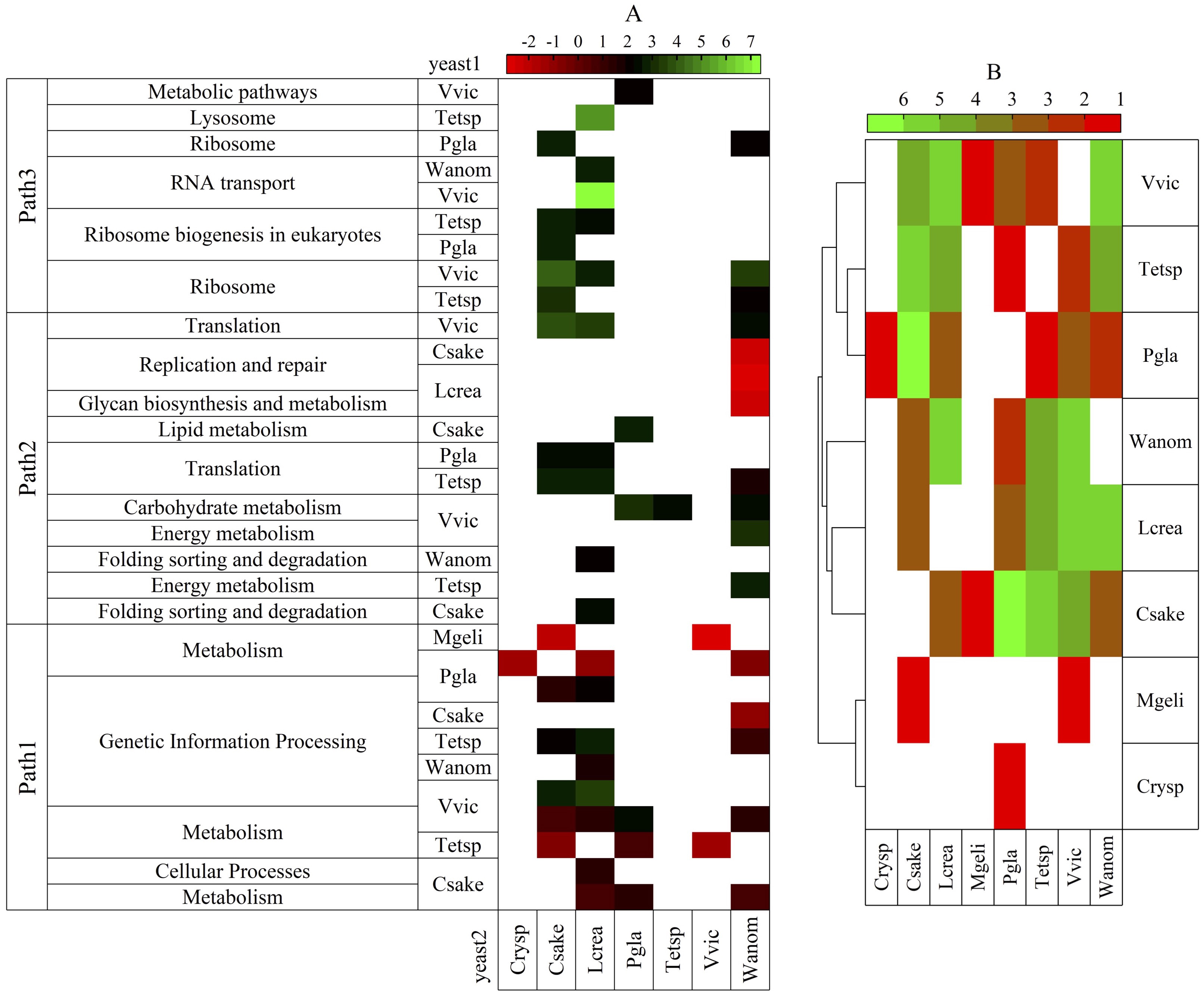

Supplement: Supplementary Figure 1 — Comparative analysis of gene expression changes among yeasts. The cellular pathways with significant differences (Tukey post hoc tests, p < 0.05) are shown. (A) Mean difference, yeast1 minus yeast2. (B) Clustering of yeasts according to the number of pathways in which they differ. [file Image_1.JPEG]

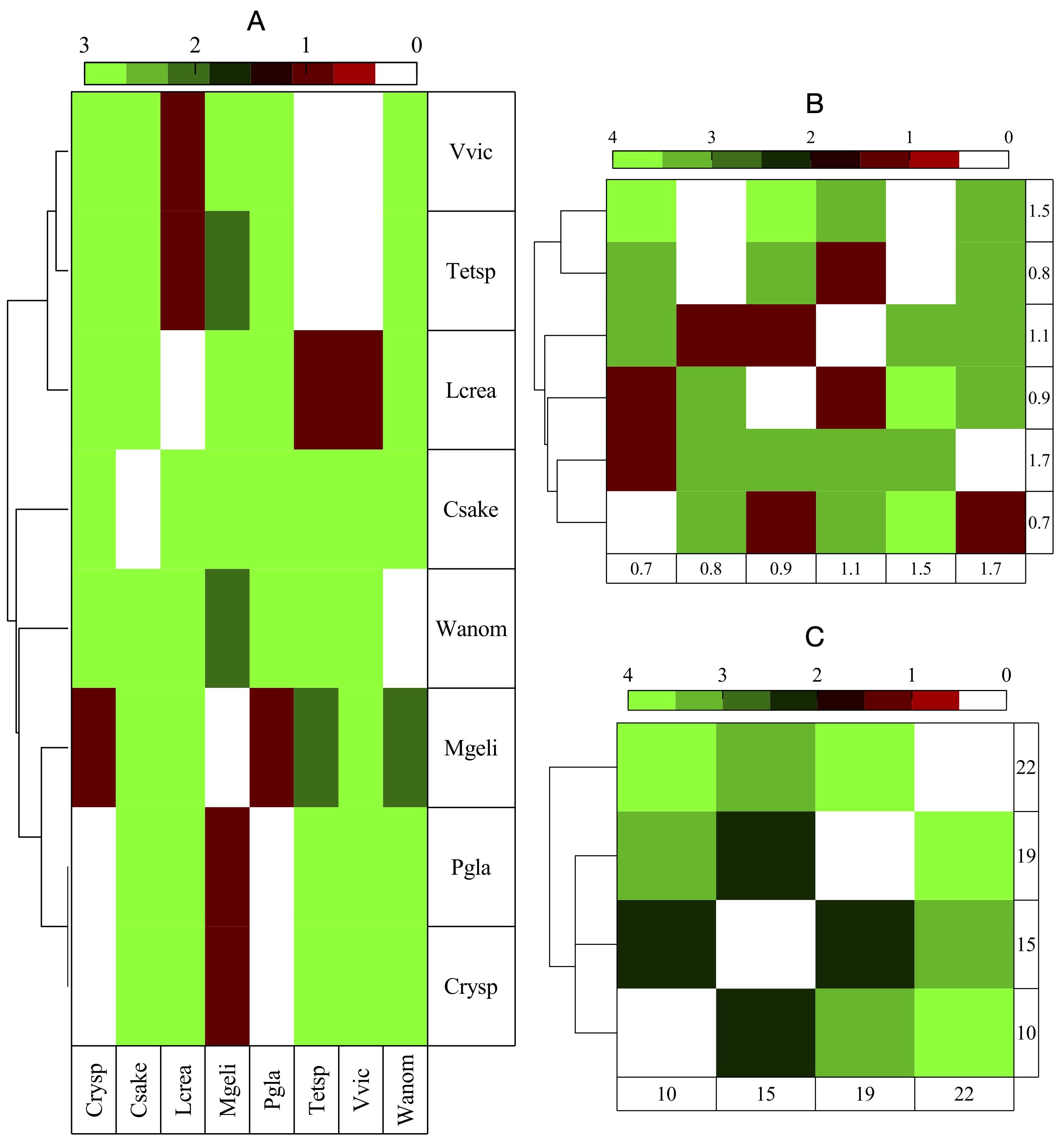

Supplement: Supplementary Figure 2 — Global comparisons of the flexibility of translated proteins among yeasts. The flexibility was estimated as percentages of very flexible (Vf) and very flexible plus moderately flexible (VMf) amino acids and class 2 (M2), and class 1 plus class 2 (M1 + 2) levels calculated by MEDUSA, compared among yeasts, and those with significant differences (Tukey post hoc tests, p < 0.05) are shown. The comparisons were performed among all yeasts individually (A) and grouped according to their growth rates (B) (h–1) and their optimal temperature for growth (C) (°C). [file Image_2.JPEG]

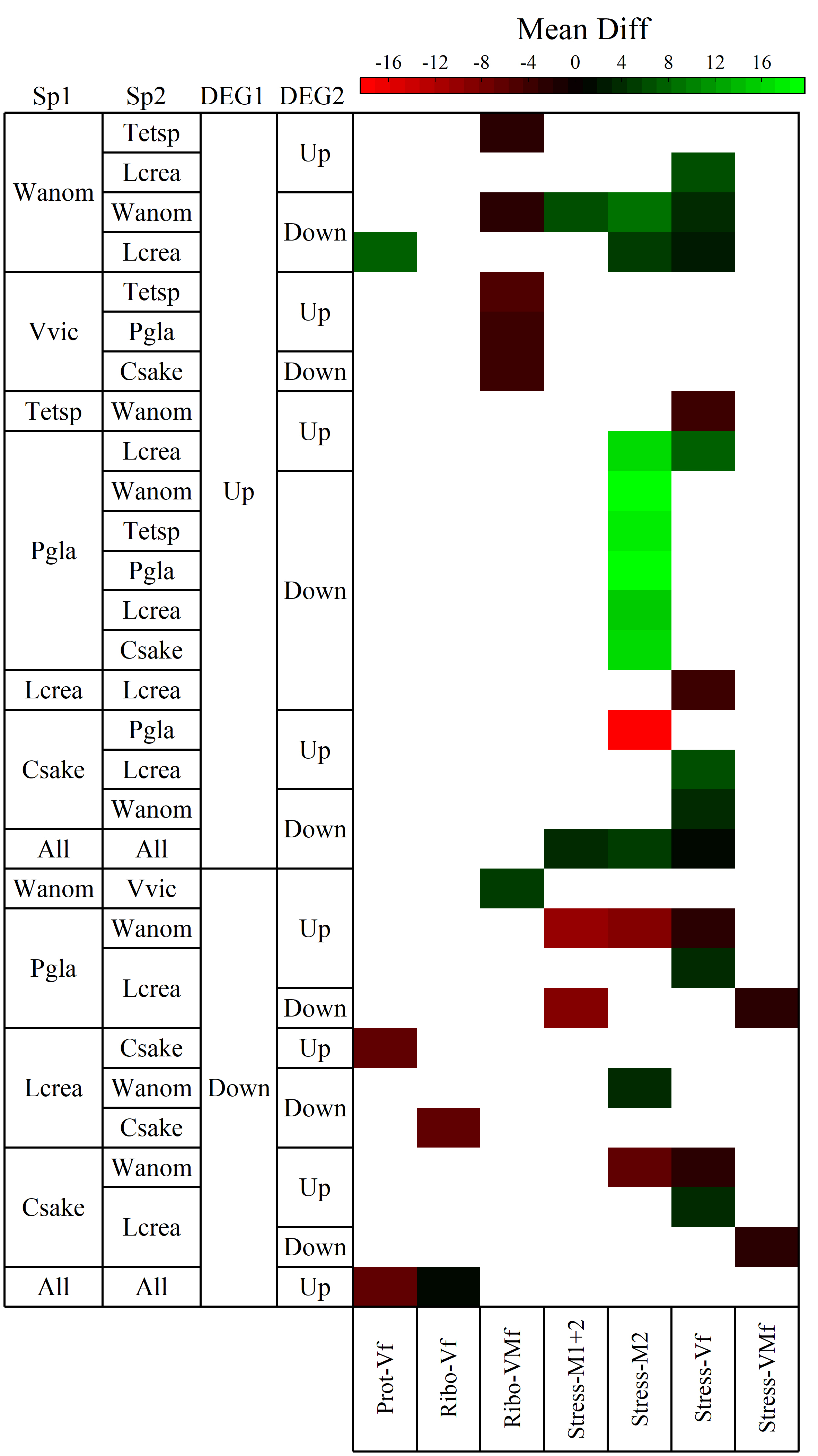

Supplement: Supplementary Figure 3 — Comparisons of the flexibility of proteins related to stress responses, proteasome subunits (Prot), and ribosomal subunits (Ribo). The proteins grouped as up- and downregulated were compared considering all and yeasts. The flexibility was estimated as percentages of very flexible (Vf) and very flexible plus moderately flexible (VMf) amino acids and class 2 (M2), and class 1 plus class 2 (M1 + 2) levels, calculated by MEDUSA. The mean difference of log2-fold change (Factor 1 minus 2) for comparisons with significant differences (Tukey post hoc tests, p < 0.05) are shown. [file Image_3.JPEG]

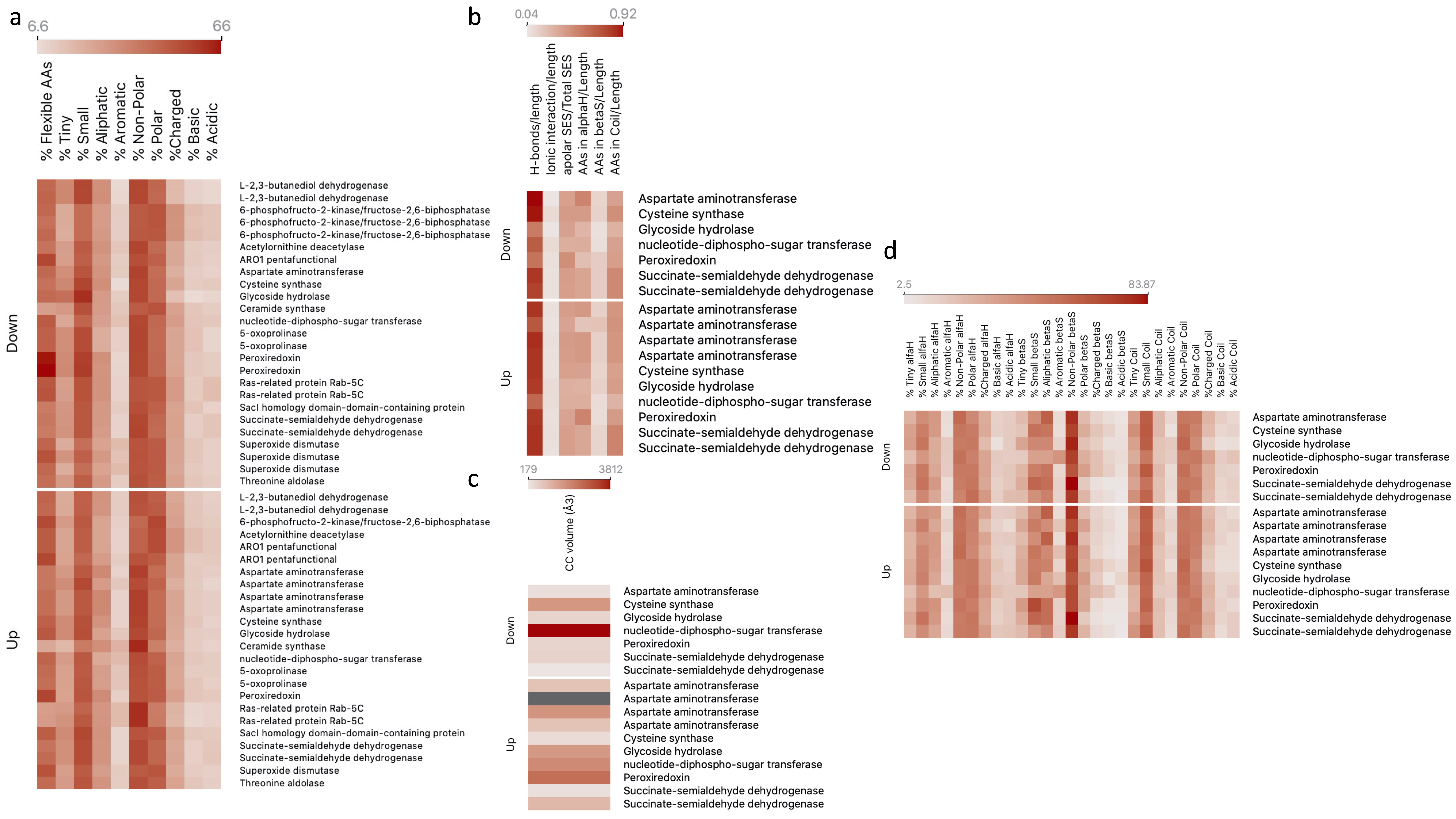

Supplement: Supplementary Figure 4 — Structural features of up- and downregulated isozymes. The structural features were calculated from 3D models constructed for each isozyme. (A) Percentages of flexibility and properties of amino acids. (B) Amino acid interactions, the fraction of apolar solvent excluding the surface (SES), and the fraction of amino acids by secondary structure. (C) catalytic site volume. (D) Percentage of amino acids having a property by secondary structures. [file Image_4.JPEG]

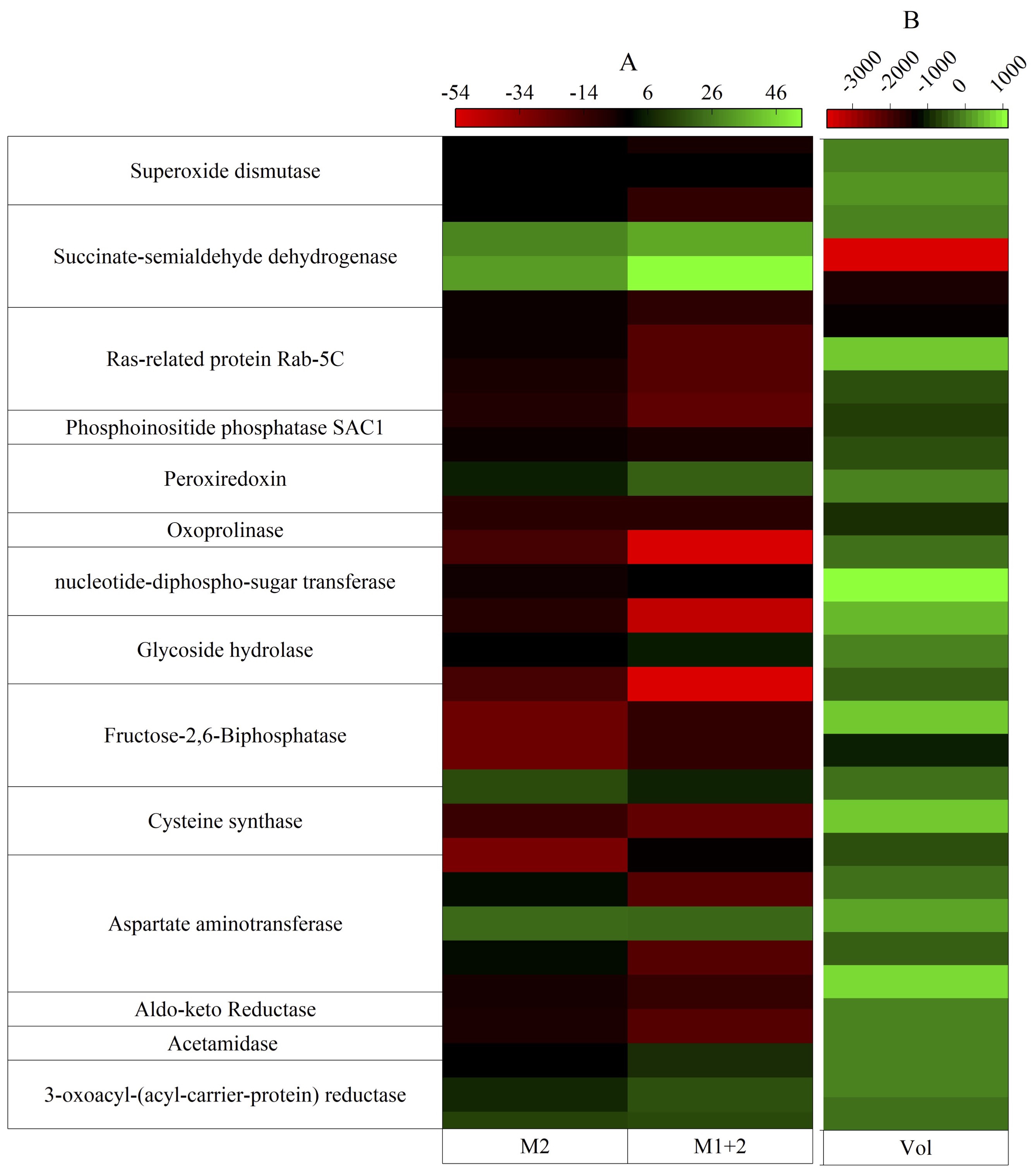

Supplement: Supplementary Figure 5 — Structural predicted active site in up- and downregulated isozymes. The structural features were calculated from 3D models constructed for each isozyme. The difference between Antarctic enzymes and mesophilic orthologs in flexibility predicted by MEDUSA (A) and the calculated volume of active site cavities (B) are shown. [file Image_5.JPEG]

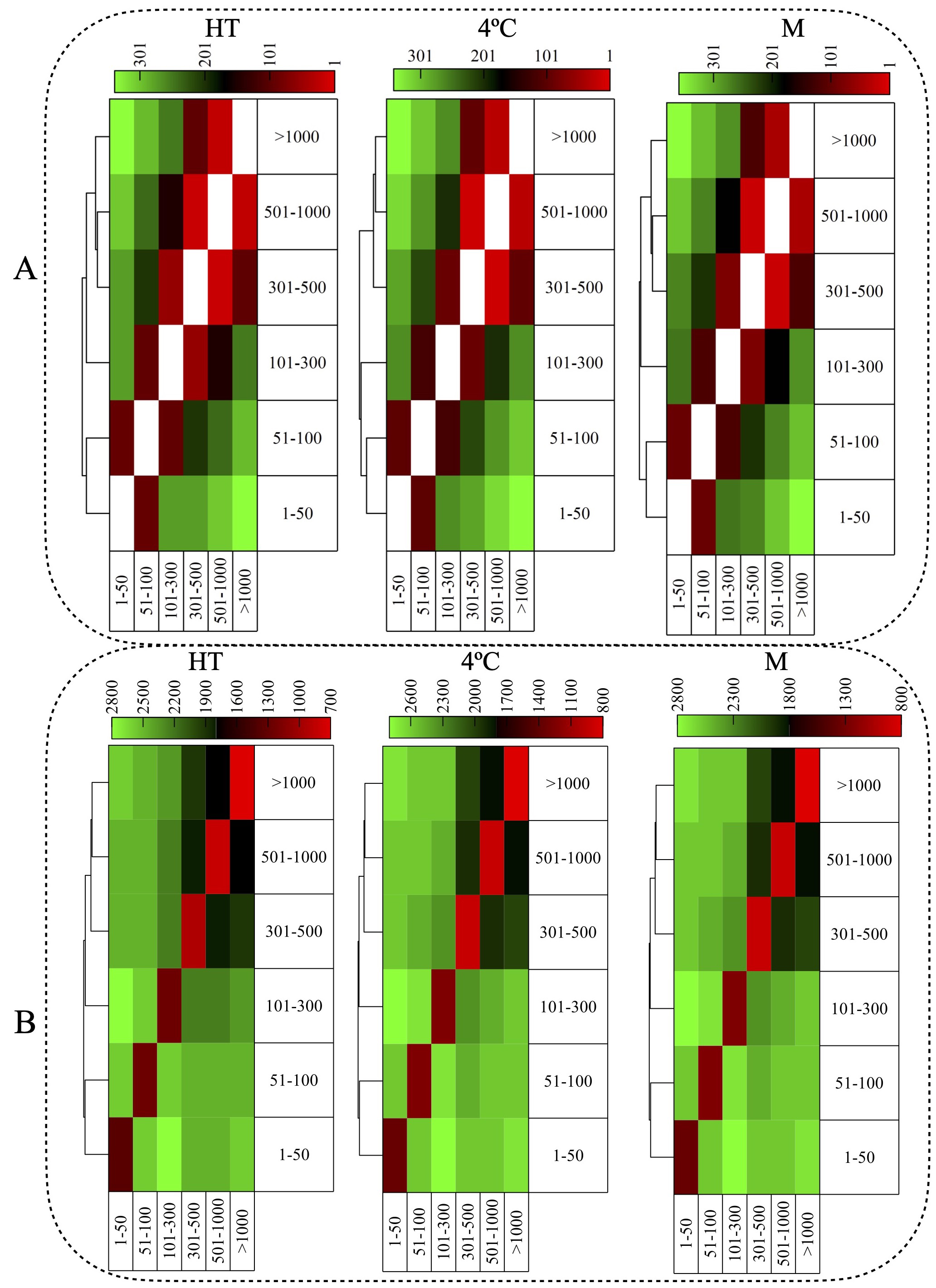

Supplement: Supplementary Figure 6 — Codon frequency comparison among putative genes grouped according to their expression levels in (A) and among (B) yeasts. The ORFs were grouped according to their RPKM values in yeasts cultivated at high temperature (HT), 4°C, and the maximum value between conditions (M). The number of codons with significant differences between groups (Tukey post hoc tests, p < 0.05) is shown. [file Image_6.JPEG]

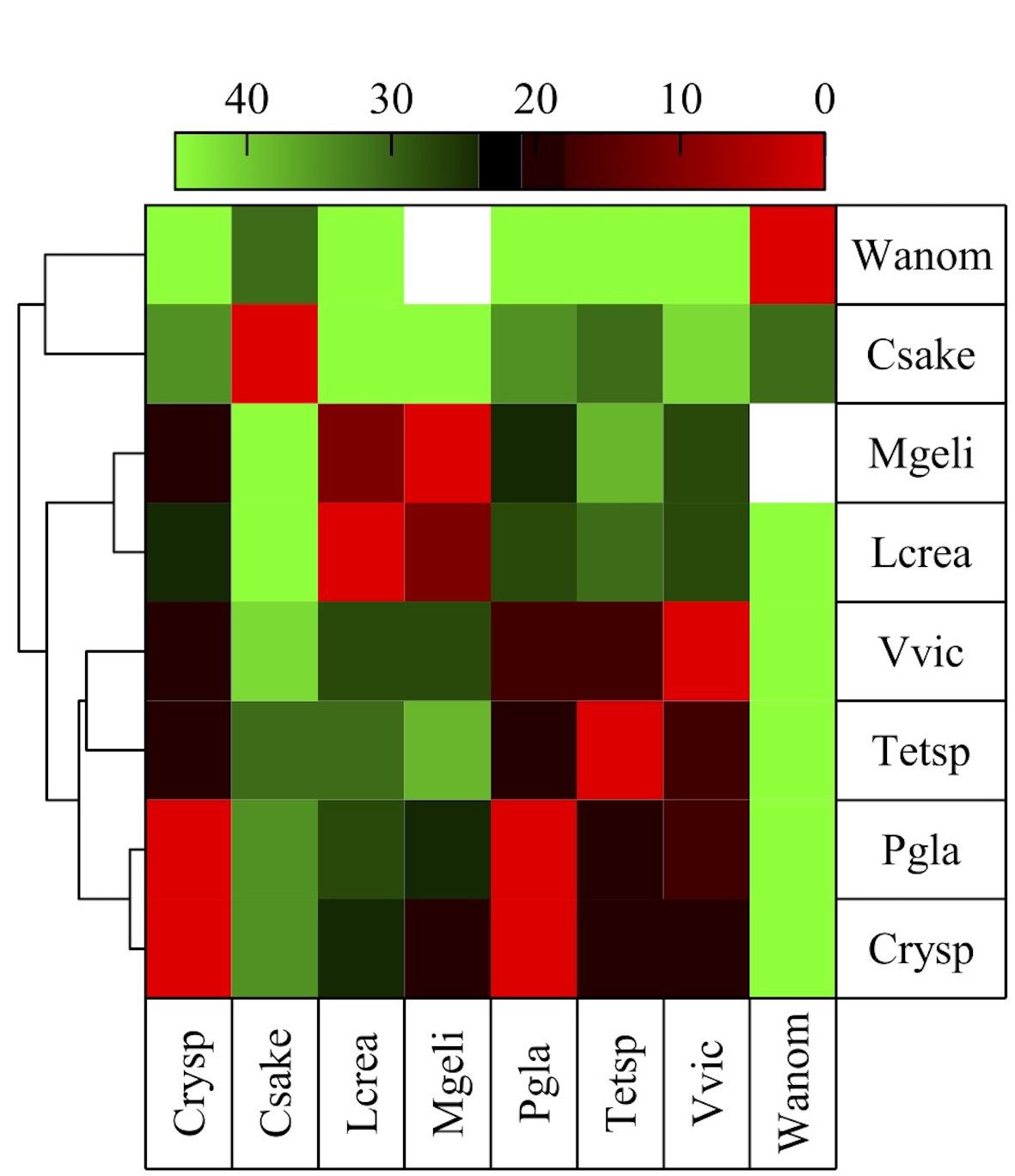

Supplement: Supplementary Figure 7 — Comparison of codon usage among yeasts considering the >1,000 RPKM group. The number of codons showing a significant difference (Tukey post hoc tests, p < 0.05) between yeast pairs is shown. [file Image_7.JPEG]
